# Anything for a cheerio: Brown capuchins (*Sapajus [Cebus] apella*) consistently coordinate in an Assurance Game for unequal payoffs

**DOI:** 10.1002/ajp.23321

**Published:** 2021-08-26

**Authors:** Lauren M. Robinson, Mayte Martínez, Kelly L. Leverett, Mattea S. Rossettie, Bart J. Wilson, Sarah F. Brosnan

**Affiliations:** ^1^ Department of Interdisciplinary Life Sciences, Domestication Lab, Konrad Lorenz Institute of Ethology University of Veterinary Medicine Vienna Vienna Austria; ^2^ Departments of Psychology, Philosophy & Neuroscience, Language Research Center Georgia State University Atlanta Georgia USA; ^3^ Economic Science Institute, Smith Institute for Political Economy and Philosophy Chapman University Orange California USA; ^4^ Departments of Psychology and Philosophy, Neuroscience Institute, Center for Behavioral Neuroscience Georgia State University Atlanta Georgia USA

**Keywords:** cooperation, economic game theory, inequality, reward value

## Abstract

Unequal outcomes disrupt cooperation in some situations, but this has not been tested in the context of coordination in economic games. To explore this, we tested brown capuchins (*Sapajus [Cebus] apella*) on a manual version of the Stag Hunt (or Assurance) Game, in which individuals sequentially chose between two options, *Stag* or *Hare*, and were rewarded according to their choices and that of their partner. Typically, coordination on *Stag* results in an equal highest payout, whereas coordinating on *Hare* results in a guaranteed equal but lower payoff and uncoordinated play results in the lowest payoff when playing *Stag*. We varied this structure such that one capuchin received double the rewards for the coordinated *Stag* outcome; thus, it was still both animals' best option, but no longer equally rewarding. Despite the inequality, capuchins coordinated on *Stag* in 78% of trials, and neither payoff structure nor their partner's choice impacted their decision. Additionally, there was no relationship between self‐scratching, a measure of stress in capuchins, and choices. After completing the study, we discovered our reward, cheerios, was sufficiently valuable that in another study, capuchins never refused it, so post hoc we repeated the study using a lower value reward, banana flavored pellets. Capuchins completed only 26% of the pellet trials (compared to 98% with cheerios), constraining our ability to interpret the results, but nonetheless the monkeys showed a decrease in preference for *Stag*, particularly when they received fewer rewards for the coordinated *Stag* outcome. These results reinforce capuchins' ability to find coordinated outcomes in the Stag Hunt game, but more work is needed to determine whether the monkeys did not mind the inequality or were unwilling to sacrifice a highly preferred food to rectify it. In either case, researchers should carefully consider the impact of their chosen rewards on subjects' choices.

## Research Highlights

− Capuchins coordinate and respond negatively to inequity.
− We tested coordination when one monkey got a greater reward for doing so.
− They consistently coordinated, but only for preferred rewards, highlighting the importance of reward value in such tasks.

## Graphical abstract

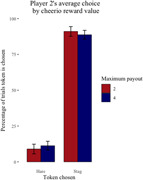

**Abbreviations**
CI: confidence interval
df: degrees of freedom
ELPD: estimated log‐predictive density
GLMM: generalized linear mixed model
LRC: language research center
SD: standard deviation
SE: standard error

## 1 INTRODUCTION

Equally contributing to achieve a shared goal does not guarantee an equally shared payoff. Dominance, chance, and other factors can make cooperation less beneficial for one party than the other, and research on inequity aversion (see reviews by Brosnan & de Waal, 2014; Oberliessen & Kalenscher, 2019) suggests that this should negatively impact cooperation. Indeed, at least some species are sensitive to inequity in the context of cooperation, with cooperation rates dropping off when one individual can dominate rewards (de Waal & Davis, 2003) or fails to share the benefits (Brosnan et al., 2006; Massen et al., 2015). Moreover, in the wild, cooperation does not lead to equal outcomes for everyone, although we do not know whether cooperation rates would differ were outcomes more equal. Nonetheless, experimental studies of cooperation, particularly in animals, often use equal payoff structures that do not reflect this reality. This is in part because the goal of these studies is typically to determine whether cooperation occurs, and so they are designed to maximize the chances of successful cooperation (hence, equal outcomes). To better understand cooperation under more ecologically realistic contexts, we need to determine how unequal pay influences the decision to cooperate.

Cooperation, as we define it here, involves interactions that, on average, provide a direct fitness benefit to the partners involved (Brosnan & Bshary, 2010; for a review of cooperation terminology see Noë, 2006). There are many examples in the animal kingdom, including group hunting (Bailey et al., 2013; Boesch et al., 2006; MacNulty et al., 2014), shared rearing of offspring (Gilchrist & Russell, 2007; Griffin et al., 2005; Russell et al., 2007), and territory defense (Farabaugh et al., 1992; Olendorf et al., 2004). Despite this prevalence, the benefits of cooperation are often not equally distributed. For example, when chimpanzees (*Pan troglodytes*) cooperate to hunt prey together, the amount of food that they obtain depends on rank, age, and other factors related to their performance in the hunt (Boesch, 1994). Indeed, while food sharing based on participation in the hunting is more common at the Taï National Park site (Boesch & Boesch, 1989; Samuni et al., 2018) than in many other chimpanzee sites (Gilby et al., 2008; Samuni et al., 2018; Watts & Mitani, 2002), nonetheless chimpanzees that perform the most important roles in the hunt take the biggest pieces of the meat (Boesch, 2002; Samuni et al., 2018). This also suggests that cooperation may succeed despite inequality, particularly in contexts in which it is justified (i.e., those who participate to a greater degree get a greater share of the outcome).

The question, then, is whether and in what contexts unequal outcomes negatively impact cooperation (Brosnan & de Waal, 2014). The link between cooperation and inequality has thus far been explored in two ways. First, from a phylogenetic perspective, multiple species respond negatively to disadvantageous inequity, or receiving less than a partner (e.g., Brosnan & de Waal, 2003; Massen et al., 2012; Wascher & Bugnyar, 2013; Yasue et al., 2018). Many species that respond negatively also routinely cooperate with nonkin in other contexts, such as group hunting or food sharing (Brosnan & de Waal, 2014), which suggests a link between cooperation and inequity. Species with less cooperative tendencies, such as orangutans (*Pongo pygmaeus*; Brosnan, Flemming et al., 2011), squirrel monkeys (*Saimiri sciureus* and *Saimiri boliviensis*; Talbot et al., 2011), and keas (*Nestor notabilis*; Heaney et al., 2017), tend not to react negatively to unequal payoffs. This supports the hypothesis, proposed initially by economists (Fehr & Schmidt, 1999), that inequity aversion and cooperation coevolved, perhaps because inequity aversion allows individuals to identify and avoid individuals that are not good cooperative partners, and specifically predicts that species that cooperate routinely should be more sensitive to inequality (Brosnan & de Waal, 2014; Brosnan, 2011; Fehr & Schmidt, 1999).

The second way that this link has been explored is by looking at the effects of unequal outcomes on cooperation. Many species are very successful at working together to achieve outcomes (i.e., cooperative tasks), such as jointly pulling a heavy tray closer, that they cannot achieve on their own. Consistent with the above, inequality often reduces the frequency or success of cooperation compared to when rewards are equal or can be shared (Campbell et al., 2020; Cronin & Snowdon, 2008; Massen et al., 2015; Melis et al., 2006). Studies of capuchin monkeys (*Sapajus [Cebus] apella*) have shown that in a bar‐pulling task, cooperation depends on the presentation of rewards and whether or how their partner shares with them. For example, capuchins tend to cooperate more with partners that share rewards (de Waal & Berger, 2000) and cooperate less when rewards are clumped, such that they can be monopolized, instead of dispersed, which makes monopolization more difficult (de Waal & Davis, 2003). When capuchins were presented with a version of the bar‐pulling task in which rewards were unequal and they could freely choose their position, the dyads that alternated which animal received the best reward did not show a decrease in cooperative outcomes (Brosnan et al., 2006); essentially they eliminated the inequity through their actions. Taken together, these results make it clear that inequity does influence capuchin cooperation, but that many animals appear to be able to use different approaches to adjust to it, including both curtailing cooperation and, more positively, reducing the inequity and thereby maintaining the cooperation.

One situation in which the impacts of inequality have not been explored is coordination in economic games. Recently, experimental economic games have been used to study various economic decisions, including cooperation, across species. Doing so allows for use of the same, or very similar, methods, thus making results more directly comparable across species, including humans (Brosnan, 2018; Watzek et al., 2018). In these games, subjects each make a decision and are rewarded depending on both their choices and that of their partner. In the simplest games, each member of a pair makes a dichotomous choice, such that there are four possible outcomes between the two players. One such game, the Assurance Game (also known as the Stag‐Hunt Game; Skyrms, 2004), is a coordination game with two strategies, *Stag* and *Hare*. Both players do better if they coordinate on *Stag* (which rewards both partners equally), but *Hare* is the risk‐free strategy because each player gets a lower payoff regardless of what their partner chooses. Both *Stag/Stag* and *Hare/Hare* are Nash equilibria, although *Stag/Stag* is payoff dominant. Uncoordinated play is the worst possible outcome because the player who plays *Stag* receives nothing. *Stag/Stag* is thus a mutual best response for both players, but if a player is unsure their partner will play *Stag*—or whether they understand the game—*Hare* is the risk‐free option.

The Assurance Game has been presented to four species of nonhuman primates, all of which have shown coordination to at least some degree, although the frequency with which they find it and the mechanism by which they do so vary. Chimpanzees have shown very high levels of understanding the game, including evidence of strategic behavior (Brosnan, Parrish, et al., 2011; Bullinger et al., 2011; Duguid et al., 2014), although they may also settle for the easy option of matching their partner, which still rewards them at very high levels (2.5 rewards per trial, on average; Brosnan, Parrish, et al., 2011; Hall et al., 2019). Rhesus macaques (*Macaca mulatta*) also do very well, playing *Stag*/*Stag*, but apparently based on a preference for the on‐average higher paying token (Parrish et al., 2014). Somewhat surprisingly, female Bolivian squirrel monkeys showed some degree of coordination, albeit less than the other species (Vale et al., 2019). Although squirrel monkeys are not generally cooperative, this is the demographic that is the most so, suggesting a need to be more specific when considering the demographics of cooperation. Finally, a few capuchins have coordinated in manual tasks, but capuchins have generally all succeeded in coordinating in computerized tasks, a difference that may be due to the increased number of trials per session and the shorter latency between choice and reward in computerized testing, both of which may support learning. However, capuchins appear to play the *Stag*/*Stag* Nash equilibrium by matching their partner's play, only succeeding when they can see what their partner has already played (Brosnan, 2011; Brosnan et al., 2012; Smith et al., 2019).

What is not known is how coordination would be impacted if their outcomes in these games were unequal. In particular, what if one subject were to receive a substantially higher payoff for playing *Stag* than the other, so it was still in both subjects' best interests to coordinate, but the subjects who got the lesser outcome would now face inequality? The situation is particularly complicated if choosing the *Hare* option meant they received absolutely less, but relatively the same. On the one hand, we know that unequal outcomes negatively impact cooperation in other contexts (such as the bar pull task), suggesting that coordination should be similarly impacted. Moreover, monkeys routinely sacrifice foods that they would typically eat to apparently protest unequal outcomes (Brosnan & de Waal, 2014), suggesting the animal receiving the lower value may choose to switch to *Hare* to avoid inequality, especially as there is no obvious reason for why one partner should get a greater reward (as in the chimpanzee group hunt). On the other hand, even the partner getting the lower value reward does absolutely better playing *Stag*, suggesting that they may still prefer to play it even if it results in a relatively less good payoff. Supporting this, in other economic games in which outcomes are not equal, such as the Hawk‐Dove game, capuchins continue to play a Nash equilibrium despite receiving less than their partner (Smith et al., 2019).

It is also important to note that just because an animal accepts an unequal outcome (for instance, continuing to play a Nash equilibrium in the Hawk‐Dove game or *Stag* in our unequal payoff Assurance Game), does not mean they are not sensitive to unequal pay. For example, dogs typically do not stop participating in a task when there is a disadvantageous difference in the reward's quality, but they show more stress behaviors (Brucks et al., 2016, 2017; Range et al., 2009) and gaze more towards their partners (Brucks et al., 2016, 2017; McGetrick et al., 2019) when the rewards are unequal. Children, too, continue to participate when receiving less, but protest to the partner (LoBue et al., 2011). Such a study has not been done in primates. However, in other contexts, primates show their frustration by performing displacement behaviors, which appear in situations characterized by psychosocial stress and include behaviors and include yawning and self‐scratching (Maestripieri et al., 1992; Troisi, 2002). In capuchin species, self‐scratching increases in response to potentially stressful situations, including receiving aggression (Daniel et al., 2009), isolation from conspecifics (Petrillo et al., 2017), and when making choices with uncertain outcomes (Sorrentino et al., 2012), suggesting that it serves a similar function. Thus, for the current study, we also measured self‐scratching behavior to see if we could detect similar behavioral differences as seen with dogs and humans even in contexts in which the capuchins accepted the inequality.

Brown capuchins are an ideal species to study the impact of unequal payoffs on coordination in the Assurance Game for several reasons. As discussed above, they naturally cooperate, even with nonkin (as reviewed by Brosnan, 2011), they are sensitive to inequality (for review see Table 1 in Brosnan & de Waal, 2014), and may punish those who benefit from unequal pay (Leimgruber et al., 2016). Additionally, capuchins coordinate in both manual and, particularly, computerized versions of the Assurance Game (Brosnan, Parrish, et al., 2011; Smith et al., 2019), which is essential for determining whether unequal outcomes negatively impact coordination. That being said, despite their increased coordination on computerized paradigms, we used a manual task rather than a computerized one because our previous work found that capuchins coordinated nearly 100% of the time in the computerized version, and we did not want to use a task for which they were already at ceiling. Finally, we displayed first players' choices (so that second players could see them) because previous work shows that seeing each other's choices is essential for capuchins to ever reach the coordinated outcome, in both manual and computerized paradigms (Brosnan, Parrish, et al., 2011; Brosnan et al., 2012; Smith et al., 2019). Unlike in previous studies, here we explicitly adopted a traditional extensive form game, controlling which player made the choice first in every session, so that the second player, who had the less valuable *Stag* outcome, always knew the partner's choice before making their own choice. We included a behavioral component, self‐scratching behavior, to explore the possibility that even if our subjects were choosing the coordinate on *Stag* despite the inequality, they found doing so to be stressful.

**Table 1:** Payout matrix for variable reward assurance game

Choice	Stag	Hare
Stag	4 or 2, 4 or 2	0, 1
Hare	1, 0	1, 1

We wanted to determine if unequal rewards and the actions of their partners impacted capuchins' choices in a cooperative task. We initially ran the experiment with cheerios (a type of oat cereal) as the reward. This is the typical reward used in our lab for all manual economic game tasks because it is both easy to manipulate and unsweetened. However, after completing the study, we discovered that cheerios are a high value reward to our monkeys that was, for many, equivalent in value to grapes (Talbot et al., 2018). This is a problem for this study as the same research also found that even the same capuchins who routinely refused rewards when their partners got better ones did not do so when they were given foods that were too high in value, and in particular, did not refuse cheerios when their partner got a greater number of them. This suggested that our results could have been due to the monkeys so strongly preferring cheerios that they would always choose the option that gave them more no matter what the partner got, even if they did care about inequity. Thus, we reran the experiment using the lowest value reward that we could find for which they would routinely exchange tokens in preference testing (banana flavored pellets) as the reward. Finally, we looked at whether self‐scratching varied depending on which option the subjects chose. Given previous studies showing capuchins continue to choose outcomes in economic games that maximized their rewards despite the fact that their partner received more (Smith et al., 2019), we predicted that capuchin monkeys would also continue coordinating in the Assurance Game, despite the unequal rewards. Additionally, we predicted that the monkeys would show more displacement behaviors (i.e., self‐scratching) in the sessions in which they received disadvantageous unequal pay.

## 2 METHODS

2.1

#### 2.1.1 Ethical approval

This study was noninvasive and approved by the Georgia State University IACUC (A16031). We additionally followed the guidelines for the Principles for the Ethical Treatment of Non‐Human Primates (American Society of Primatologists, 2001).

#### 2.1.2 Data availability

These data have been made publicly available in Data S1 and S2 and in an online repository (https://osf.io/h7emb/).

#### 2.1.3 Subjects

We studied six pairs (1: M/M, 1: F/F, 4: M/F) of capuchin monkeys ranging from 9 to 20 years old, drawn from social groups housed at Georgia State University's Language Research Center (LRC) in Atlanta, Georgia; information on each animal's sex and group can be found in Table S1. Subjects came from one of five mixed‐sex socially house capuchin groups at the facility, each of which has its own indoor/outdoor enclosure with enrichment and climbing structures. Subjects had been housed in the same social group since either 2005 or their birth at the LRC. Capuchins at the LRC are never deprived of food for testing purposes and water is available at all times, including during testing sessions.

All studies at the LRC are noninvasive and subjects voluntarily choose to participate. Subjects are never deprived of food, water, treats, outdoor time, or social contact to encourage participation, but could choose to enter individual test boxes where the experiment took place. There was no consequence for choosing not to participate other than not being able to participate in the activity. Upon the completion of testing (approximately 30 min), the subjects were released back into their social group. All subjects had previously been trained to exchange tokens for food rewards.

#### 2.1.4 Unequal assurance game

In the Assurance Game individuals must decide whether to coordinate for a large reward that is dependent on their partner's choice or not coordinate for a guaranteed smaller reward. In the traditional game, *Stag*/*Stag* is the payoff‐dominant Nash equilibrium.^1^ Playing *Hare* results in a sure smaller reward; hence, *Hare*/*Hare* is the risk‐dominant Nash equilibrium. Playing *Stag* when the partner plays *Hare* brings no reward at all, making the uncoordinated outcome the worst possible outcome for the individual who chooses *Stag*. In our case, we were interested in their decisions when the *Stag*/*Stag* equilibrium gave the two players different rewards; in both cases, subjects got more by playing mutual *Stag* than any other possibility, but one partner got half as many rewards (2 vs. 4) as compared to their partner (see Table 1 for a payoff matrix).

#### 2.1.5 Quantity testing

All subjects' preferences were previously tested on different quantities of cheerios and all significantly preferred four cheerios over two cheerios and two cheerios over one cheerio (Leinwand & Brosnan, unpublished data). Subjects were given a choice between the two quantities, alternating the side on which each was presented, and were able to eat whichever one they picked. To meet criterion, subjects had to prefer the greater number of cheerios to the fewer on at least 8 out of 10 trials on each of two separate days.

#### 2.1.6 Testing procedure

First, the subjects were called into individual test boxes that were attached to the indoor section of their group's home enclosure, situated side by side. Test boxes were 18″ apart (hence subjects were separated by that distance from their partner). Subjects were only tested with other members of their established social group. Each test box was outfitted with a clear Lexan door with two holes large enough for the monkey to reach through to the container containing the two types of tokens (*Stag* and *Hare*). The tokens were approximately 1″ diameter three dimensional hexagons printed in food safe MakerBot brand filament using a MakerBot 3D printer. The *Stag* token was orange in color while the *Hare* token was black in color, however because capuchins are often dichromats, the *Hare* token also included one white spot on each of the hexagon faces while the *Stag* token was solid in color to offer an additional method to discriminate the options.

For each trial, each subject made their choice from their own container of tokens, which always included three *Stag* tokens and three *Hare* tokens. The experimenter shook the container, before presenting it to the subject, to ensure the tokens were randomly arranged in the container; this choice mechanism was used to avoid the side biases common in primate studies. The subject designated as Player 1 was given the option to choose their token first. Once Player 1 chose a token, that monkey handed it back to the experimenter, who took it, held it in front of the Lexan door so that both individuals could see it, and then placed it on the piece of paper we used to increase visual contrast and emphasize the chosen token. This was located between the two monkeys on the testing cart that held the choice containers, in view of both subjects. This procedure was then repeated for Player 2. Following Player 2's token exchange, the subjects were simultaneously rewarded with cheerios according to both subjects' choices, one cheerio at a time to maximize the likelihood that they saw both what they and their partner received (e.g., for coordinated *Stag* play, the experimenter would hand both subjects a cheerio, followed by the second, and so forth, whereas for uncoordinated play, only the subject who chose *Hare* got a single cheerio). Once the subjects finished eating their food rewards, the next trial began.

One key difference between this test and our previous work is the explicitly sequential choice of the partners. In previous work, both in manual (exchange‐based) and computerized versions of the task, subjects were presented with their choices simultaneously (and in some versions of the computerized task, they did not know what their partner had chosen until they had made their choice; Brosnan, Flemming et al. 2011; Smith et al., 2018). In our case, however, the sequential choice was essential so that the second player could make their choice based on what the first player chose. Although this changes the structure of the game from prior work, we note that in our earlier work subjects would only play the coordinated Nash equilibrium reliably when they could see their partner's choice anyway, suggesting that they habitually play the game sequentially, making their choice based on their partner's play. All sessions were videotaped for further analysis, although 12 sessions are missing due to recorder malfunctions. In those cases, analysis was based on experimenter coded data, and we did not include those sessions in analyses of self‐scratching (because those data were not coded by the experimenter in real time).

#### 2.1.7 Lower value food test

We initially planned to run the study using only the cheerios breakfast cereal as the food reward. However, subsequent to the completion of testing, another study we completed with the same monkeys found that subjects that would refuse lower value foods when their partner got a better one did not refuse high value foods, even if their partner did get an even more preferred one. Moreover, cheerios were very highly valued; many subjects preferred them as highly as grapes and no subject consistently refused them in a quantity based inequity test in which their partner got a greater number of cheerios, even though some of the same monkeys refused lower‐value foods if their partners got more preferred ones (and all monkeys had passed quantity preference tests demonstrating that they preferred more to fewer cheerios in a direct dichotomous choice; Talbot et al., 2018). Because this suggested that our results could simply be due to monkeys' unwillingness to refuse cheerios, regardless of what their partner received, we decided, post hoc, to repeat the study using a lower value food.

Unfortunately, it was challenging to find a food that all subjects considered lower value than cheerios and for which the capuchins would consistently work. Eventually we settled on Bio‐Serve precision banana flavored pellets, their typical reward in computerized tasks and one for which they would exchange tokens. A preference test (using the same procedure outlined above) demonstrated that all but one subject preferred cheerios to pellets 100% of the time, and the exceptional subject preferred cheerios to pellets 90% of the time. Once testing was underway, however, most subjects began refusing to work for the pellets and no pair completed all of these sessions. Because these results shed light on the hypothesis that the food value influenced responses to the task, we analyzed and present this partial data set, but emphasize that these results cannot be taken as conclusive. Nonetheless, we think it is important to discuss the challenges inherent in finding appropriate rewards for cognitive and behavioral experiments.

#### 2.1.8 Testing schedule

The goal was for each pair to participate in 20 sessions consisting of 40 trials for each reward type, although, again, no subject completed testing with the pellets. No subject participated in more than one session per day and subjects were generally tested 2–3 days per week. The 20 sessions were separated into four blocks (i.e., four blocks of five sessions each) so that each subject played in each of the four possible combinations of being Player 1 or Player 2 and reward payout for choosing *Stag*. The payout for *Hare* remained the same throughout the study. The block schedules were counterbalanced such that three players began with Player 1 receiving the *Stag* payout of two cheerios while the other three pairs began with Player 1 receiving a *Stag* payout of four cheerios. Because we ran the pellet study to address a post hoc question that emerged after the completion of the original round of testing, all subjects completed all sessions with cheerios as a reward first before running the sessions with pellets as the reward.

#### 2.1.9 Behavioral coding

All sessions were videotaped, however as noted above, we lost 12 sessions due to equipment failure and one dyad failed to complete two sessions. For the remaining cheerio sessions, we coded the number of self‐scratching bouts across a session using Loopy (http://loopb.io; Loopbio GmbH). Self‐scratching was defined as “the focal (i.e., observed) capuchin moves its hand or foot rapidly drawing its fingers/fingernails, toes/toenails, or the back of the hand across the hair or skin; when the hand or foot stops moving the event is done.” In some videos, one or both animals were difficult to see, thus making it difficult to get an accurate self‐scratching count. Therefore, we only coded videos where both animals were clearly visible, this left us with 83 coded sessions across the six dyads. Six volunteers coded how long a session lasted and the frequency of self‐scratching for each animal in a dyad across the entire session,

Although the common convention regarding interobserver reliability is to randomly select 20% of the observations to second code, this is not supported by any statistical reason for doing so. Arguably, by randomly selecting 20% of the videos, one may not get an equal representation of the data across the chosen videos. For example, the videos with the highest frequency of a behavior, in this case scratching bouts, may not be randomly selected and, as such, reliability is only assessed when scratching is less frequent. Therefore, we took an alternative approach: We first constructed a series of eight integer numbers spanning from the minimum to the maximum number of scratching bouts per video (rounding ensured that they were all integers). We then selected one video for each of these targeted numbers, whereby we chose the one with a number scratches as close as possible to the targeted value (if several were equally close, we randomly selected one of them). By doing so we made sure that the range of the number scratches was roughly evenly represented in the videos to be recoded. This resulted in eight videos to be coded a second time. This reliability coding was performed by two of the volunteers, neither of whom coded any videos they coded the first time. We found interobserver agreement of *ρ* = 0.91 for counts of self‐scratching bouts across the sessions, suggesting the scratching data was appropriately reliable for statistical analysis.

### 2.2 Data analyses

All statistics were performed using R version 4.0.0 (R Core Team, 2020).

#### 2.2.1 Cheerios analyses

To model the influence of the partner's choice (i.e., *Stag* or *Hare*) and reward maximum reward payout (i.e., player received two or four cheerios for choosing *Stag*) we used Generalized Linear Mixed Models (GLMM; Baayen, 2008) with a binomial error structure and logit link function using the glmer function in lme4 package in R (Bates et al., 2015).

To investigate if Player's 1 choice to coordinate (i.e., if they selected *Stag*) and the reward structure (i.e., did they receive two or four cheerios for coordinating on *Stag*) influenced Player 2's choice to coordinate (i.e., if they selected *Stag*), we included Player 1's choice, Player 2's maximum reward payout for the trial, trial number, block number (i.e., which of the four blocks the trials took place within), and their four‐way interaction, including all the terms this encompassed, as fixed effects. We included block number and trial number as fixed effects because we expected the capuchins to learn the reward structure and adjust their choices in response to their partner's choice as they participated in more trials and blocks. We included the random intercepts of Player 2's identity and dyad identity to control for the influence of identity (individual and dyad) on choice as one individual or dyad may have been more cooperative than another. We included the four‐way interaction, and all the terms it encompassed, of Player 1's choice, trial number, block number, Player 2's maximum reward payout as random slopes to keep the Type I error rate within 5% (Barr et al., 2013; Schielzeth & Forstmeier, 2009). However, we found most of the absolute correlations to be close to 1, indicating they were unidentifiable (Matuschek et al., 2017) and thus, added little to the model while making it more complex. Therefore, following this, we removed the correlations, resulting in a relatively minor change to model fit (log‐likelihood with correlations: −1263.65, *df* = 288; log‐likelihood without correlations: −1282.20, *df* = 48).

To investigate if Player 2's previous choice to coordinate (i.e., if they chose to play *Stag* in the previous trial) and the reward structure (i.e., did they receive two or four cheerios for coordinating on *Stag*) influenced Player 1's choice to coordinate (i.e., if those chose *Stag*), we included Player 2's previous choice, Player 1's maximum reward payout, trial number, block number, and their four‐way interaction, including all the terms this encompassed, as fixed effects. For the same reasons as in the first model (i.e., learning), we included block number and trial number as fixed effects. We included the random intercepts of Player 1's identity and dyad identity to control for the influence of identity (individual and dyad) on choice, as in the first model, because one individual or dyad may have been more cooperative than another. We included the four‐way interaction, and all the terms it encompassed, of Player 2's previous choice to cooperate, trial number, block number, and Player 1's maximum reward payout as random slopes, again to keep the Type I error rate within 5% (Barr et al., 2013; Schielzeth & Forstmeier, 2009). As in the previous model, we started by fitting the model, including the correlations between the random slopes. Again, they were close to 1 and a loglik comparison between the model with and without correlations revealed a very minor change in the model fit. Thus, we then removed the correlations (log‐likelihood with correlations: −1452.90, *df* = 288; log‐likelihood without correlations: −1472.23, *df* = 48).

To test the effect of player choice and reward structure in the models, we conducted full‐null model comparisons (Forstmeier & Schielzeth, 2011) where the variables of interest are removed and the model is compared to the full model to test if patterns in the data are the result of random sampling (i.e., the full model is not significantly different from the null model) or not (i.e., the full model is significantly different from the null model; Gotelli & Ulrich, 2012). We did this to avoid cryptic multiple testing (i.e., starting with the full model and removing nonsignificant variables from each model until there are only significant variables left, thus increasing p‐inflation from increased numbers of models; Forstmeier & Schielzeth, 2011) using a likelihood ratio test (Dobson & Barnett, 2008). The null models were identical to the full models except we removed player choice and the player's maximum reward value, and also all the interactions they were involved in, from both models. For all models, we z‐transformed trial number and block number to aid model interpretation (Schielzeth, 2010) and before including it as a random slope, we z‐transformed the player's maximum reward payout to ease model convergence. We calculated the confidence intervals of model estimates using a parametric bootstrap (*N* = 1000 bootstraps).

Finally, to investigate if the reward structure influenced self‐scratching behavior in the cheerio sessions, we took multiple steps. First, we tested the interobserver reliability of our behavioral coding using a Spearman's rank correlation. Following this, we fit a GLMM with Poisson error distribution and log link function predicting the number of scratches an animal performed across the session. As the fixed effects, we included the interaction of block number and player's maximum reward; both block number and maximum reward payout was z‐transformed. We included the interaction of block number and maximum reward payout for coordinating on *Stag/Stag* as random slopes within the random intercept of subject ID. As an offset variable (McCullagh & Nelder, 1989), we included the duration of the session, from the first animal's choice to the last animal's choice, which we log transformed. Due to a problem with overdispersion with the Poisson error distribution (dispersion parameter = 5.98), we switched to a negative binomial distribution, which showed the model not to be over dispersed (dispersion parameter = 1.08) and therefore, we interpreted the results of the negative binomial model. As before, we performed a full null‐model comparison using the same distribution; the null model consisted of the same variables except maximum reward payout was removed.

#### 2.2.2 Pellet analyses

As mentioned above, we reran the study with pellets as a food reward due to the possibility that the capuchins never refused the cheerios because they were too high value (i.e., Talbot et al., 2018). Although they initially consistently worked for our lower value food, pellets, which met our criterion (cheerios preferred by every subject on more than 80% of trials on two consecutive sessions), most monkeys rapidly ceased working for pellets and no pair completed all tests. Thus, we analyze these data but interpret the results cautiously, given the incomplete nature of the data set.

To investigate if their partner's choice to coordinate (i.e., if they chose to play *Stag* in the previous trial) and the maximum reward payoff (i.e., did they receive two or four cheerios for coordinating on *Stag*) influenced Player 1 and Player 2's choice to play *Stag* when pellets were used as rewards, we first fit two GLMMs with Poisson error distribution.

In the first Poisson model, we included the number of times that Player 1 chose *Stag* as the response variable. We included Player 2's choice in the previous trial and Player 1's maximum reward payout, and their two‐way interaction, as fixed effects. As an offset variable, we included the total number of times Player 1 made a choice within each parameter (e.g., the total number of Player 1 choices when Player 2 chose *Stag* in the previous session and the animal's maximum payout was two pellets). We included the random intercepts of Player 2's identity and dyad identity because, as in the analyses of the data with cheerios as the reward, one individual or dyad may have been more cooperative than another.

In the second Poisson model, we included the number of times that Player 2 chose *Stag* as the response variable. We included Player 1's choice and Player 2's maximum reward payout, and their two‐way interaction, as fixed effects. As an offset variable, we included the total number of times Player 2 made a choice within each parameter (e.g., the total number of Player 2 choices when Player 1 chose *Stag* and the animal's maximum payout was two pellets). We included the random intercepts of Player 1's identity and dyad identity to account for the possibility that one individual or dyad was more cooperative.

Second, to investigate whether payoff reward (i.e., cheerios or pellets) influenced the animals' choice to coordinate, we conducted informed Bayesian regressions with change points. We switched to a change point analysis because the lack of counterbalancing in the pellet data did not allow us to directly compare them to the cheerio data using standard GLMMs, whereas change point analyses are used to detect when a trend changes in sequential data. We followed a Bayesian approach that models the probability of the parameters in the model taking into account both prior beliefs (i.e., “priors”) on the distribution and mean of a variable and the resulting probability once the actual data are introduced in the model (McElreath, 2018). Using the mcp package in R (Lindeløv, 2020), we tested if the proportion of *Stag* choices for Player 1 and Player 2 changed when the reward used were pellets (Session 21). As a first step, we built an “empty” model (i.e., without data) consisting in one regression line followed by a disjoined changing rate. As a prior belief, we indicate that we expected a change in performance around Session 21. For the rest of parameters, we used the default uninformative priors for a model with one change point provided by the package (Dirichlet prior; Bürkner & Charpentier, 2018).

Following this, we ran two sets of Generalized Linear Models with binomial distribution, using either the proportion of *Stag* choices for Player 1 or Player 2 as variable response and the session number as predictor. Every set consisted in three models, one informed model using the priors that we defined before (expected change around Session 21), one uninformed model using the default priors, and one null model that did not include any change point. We checked the convergence of all the models using the Gelman–Rubin convergence diagnostic. All the parameters were below 1.1, which indicates good convergence (Gelman & Rubin, 1992).

Once we fit all the models, we tested the existence of the change point and the contribution of the priors in our informed model by comparing the predictive performance of the informed, uninformed and null model in terms of their estimated log‐predictive density (ELPD). To calculate the ELPD, the function “loo” fits the model to the data multiple times, each time lacking one of the data points. ELPD is obtained with the average log‐likelihood of all the models, indicating how well the model is able to predict the data points that are lacking (Vehtari et al., 2017). Therefore, the model with higher ELPD shows a superior predictive performance. We selected the model with higher ELPD for the *Stag* choices of Player 1 and Player 2 to test the hypothesis of the change point being between the Session 20 and 21. For this, we computed the Bayes Factor via Savage–Dickey density ratio and posterior contrast (Verdinelli & Wasserman, 1995), to assess if our belief in the location of the change point is stronger after fitting the data with the actual data.

## 3 RESULTS

### 3.1 Cheerio results

**Figure 1:**
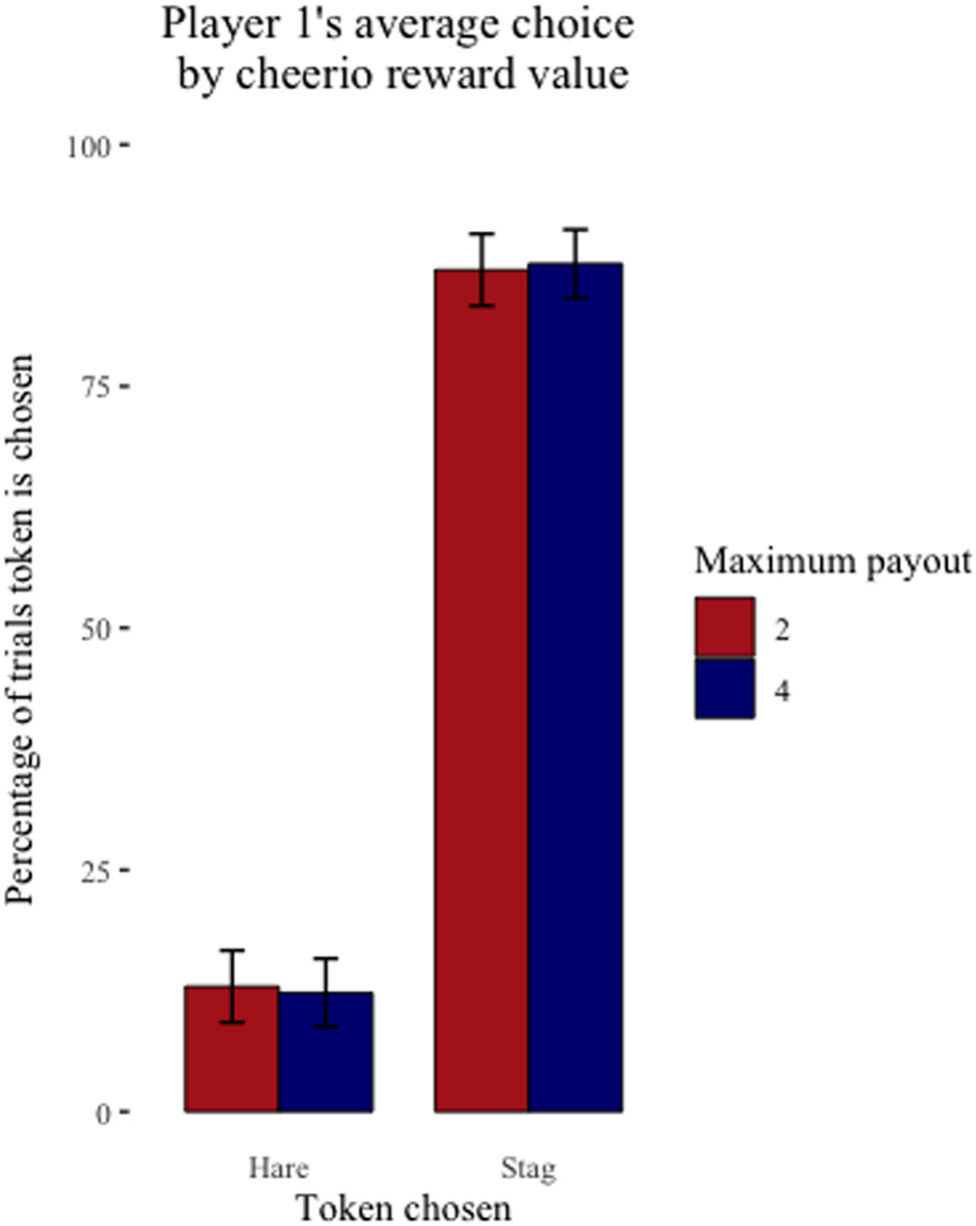
Player 1's average individual choice (Stag or Hare) ± SE by their maximum payout for coordinating on Stag/Stag when cheerios are the reward

**Figure 2:**
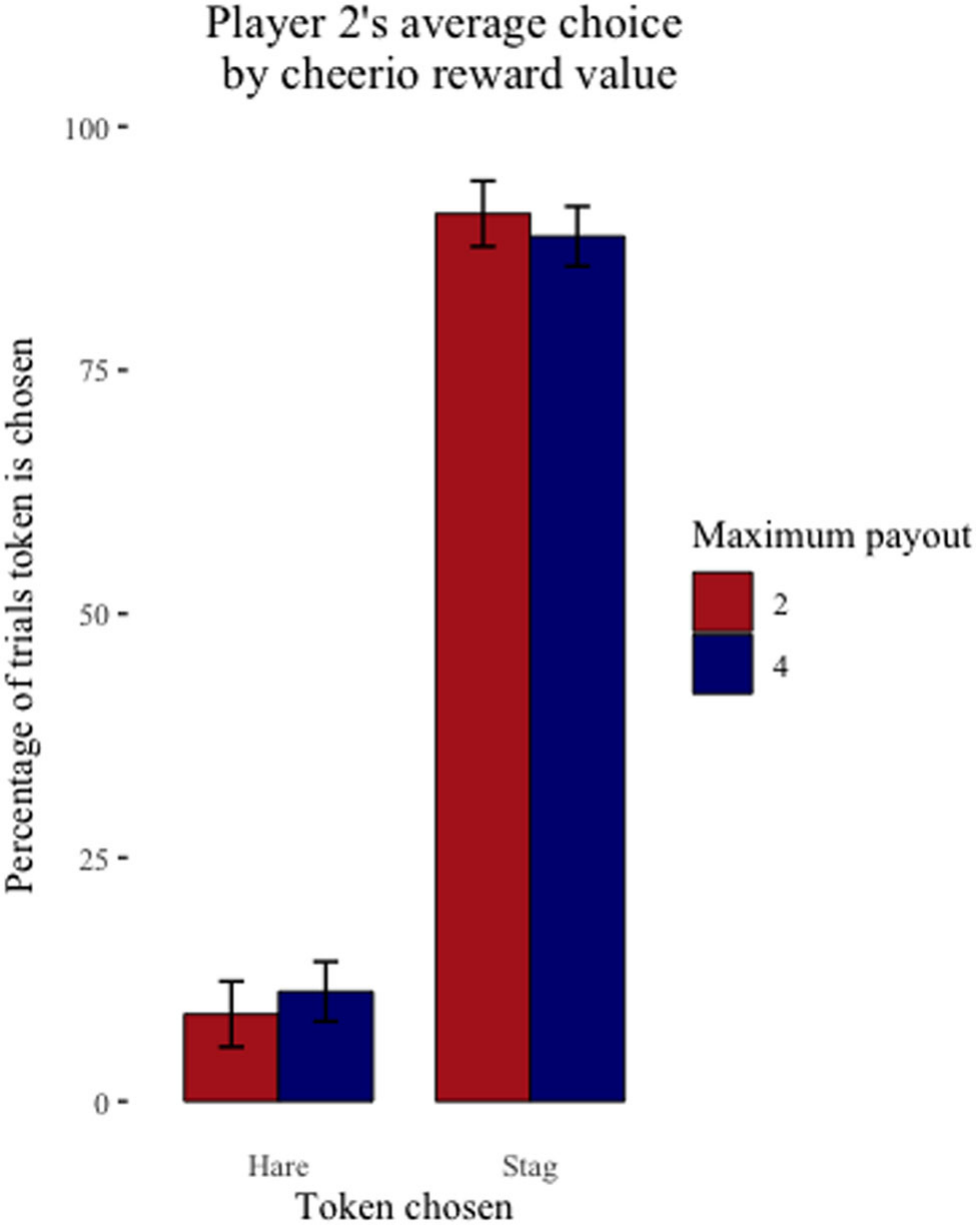
Player 2's average individual choice (Stag or Hare) ± SE by their maximum payout for coordinating on Stag/Stag when cheerios are the reward

Across the sessions where cheerios were the reward, the six capuchin dyads completed 4719 trials over 118 sessions (one dyad chose not to complete two sessions) with cheerios as the reward. Of these trials, they chose to coordinate on the payoff dominant Nash equilibrium (*Stag*/*Stag*) 3707 (78%) times and the risk dominant Nash equilibrium (*Hare*/*Hare*) 79 (2%) times and chose the uncoordinated outcome (*Stag*/*Hare* or *Hare*/*Stag*) 933 (20%) times. On average, Player 1 chose *Stag* over *Hare* 87% of the time when their maximum payout was two cheerios and 88% of the time when their maximum payout was four cheerios (Figure 1). On average, Player 2 chose *Stag* over *Hare* 91% of the time when their maximum payout was two cheerios and 89% of the time when their maximum payout was four cheerios (Figure 2).

#### 3.1.1 Model predicting Player 2's choice

Considering the model testing if Player 2's choice of *Stag* was influenced by Player 1's choice and Player 2's maximum reward (i.e., two or four cheerios for a *Stag*/*Stag* outcome), we found this model (Table 3; estimated SD of random effects of model in Table S2) was not significantly different from the null model (likelihood ratio test comparing full and null model: *χ* ^2^ = 13.13, *df* = 12, *p* = 0.36), suggesting that variables where *p* < 0.05 were likely due to Type I error due to multiple testing. There was also no significant effect of the four‐way interaction of Player 1's choice, Player 2's maximum reward payout, trial number, and block number (*b *= 0.34, SE = 0.20, *p* = 0.084). In summary, there was no effect of Player 1's choice or Player 2's maximum reward payout on Player 2's choice.

**Table 2:** Testing block player and payout schedule for variable reward assurance game

Block 1	Player 1	Player 2	Block 1	Player 1	Player 2
Dyad	Stag = 2	Stag = 4	Dyad	Stag = 4	Stag = 2
Lily/Wren	Lily	Wren	Mason/Gonzo	Mason	Gonzo
Griffin/Widget	Widget	Griffin	Benny/Gretel	Benny	Gretel
Nkima/Nala	Nala	Nkima	Liam/Logan	Liam	Logan

**Block 2**	Player 1	Player 2	**Block 2**	Player 1	Player 2
Dyad	Stag = 4	Stag = 2	Dyad	Stag = 2	Stag = 4
Lily/Wren	Wren	Lily	Mason/Gonzo	Gonzo	Mason
Griffin/Widget	Griffin	Widget	Benny/Gretel	Gretel	Benny
Nkima/Nala	Nkima	Nala	Liam/Logan	Logan	Liam

**Block 3**	Player 1	Player 2	**Block 3**	Player 1	Player 2
Dyad	Stag = 2	Stag = 4	Dyad	Stag = 4	Stag = 2
Lily/Wren	Wren	Lily	Mason/Gonzo	Gonzo	Mason
Griffin/Widget	Griffin	Widget	Benny/Gretel	Gretel	Benny
Nkima/Nala	Nkima	Nala	Liam/Logan	Logan	Liam

**Block 4**	Player 1	Player 2	**Block 4**	Player 1	Player 2
Dyad	Stag = 4	Stag = 2	Dyad	Stag = 2	Stag = 4
Lily/Wren	Lily	Wren	Mason/Gonzo	Mason	Gonzo
Griffin/Widget	Widget	Griffin	Benny/Gretel	Benny	Gretel
Nkima/Nala	Nala	Nkima	Liam/Logan	Liam	Logan

**Table 3:** Player 2's choice of Stag predicted by Player 1's choice and Player 2's maximum reward payout

Term	*b*	SE	CI	*p*
Intercept	4.66	0.86	(3.51, 6.35)	
Player 1's choice (Stag)	−1.40	0.77	(−2.74, −0.42)	0.072
Trial number^a^	0.40	0.75	(−0.72, 1.49)	0.59
Block number^b^	1.04	0.94	(−0.51, 2.73)	0.27
Player's 2 maximum payout	−0.55	0.22	(−0.96, −0.18)	**0.014**
Player 1 choice (Stag): Trial number	−0.31	0.75	(−1.40, 0.80)	0.68
Player 1 choice (Stag): Block number	0.03	0.72	(−1.12, 1.08)	0.96
Trial number: Block number	1.59	0.74	(0.47, 2.74)	**0.032**
Player 1 choice (Stag): Player 2's maximum payout	0.47	0.22	(0.13, 0.84)	**0.034**
Trial number: Player 2's maximum payout	−0.06	0.22	(−0.41, 0.28)	0.77
Block number: Player 2's maximum payout	−0.09	0.28	(−0.62, 0.45)	0.75
Player 1 choice (Stag): Trial number: Block number	−1.19	0.68	(−2.23, −0.24)	0.082
Player 1 choice (Stag): Trial number: Player 2's maxium payout	0.12	0.22	(−0.22, 0.45)	0.60
Player 1 choice (Stag): Block number: Player 2's maxium payout	0.02	0.20	(−0.32, 0.36)	0.92
Trial number: Block number: Player 2's maxium payout	−0.44	0.22	(−0.82, −0.09)	0.044
Player 1 choice (Stag): Trial number: Block number: Player 2's maxium payout	0.34	0.20	(0.03, 0.68)	0.084

*Note:* Bold values were significant at *p* < 0.05.

Abbreviation: CI, confidence interval.

^a^ z‐Transformed to a mean of zero and a standard deviation (SD) of one; mean and SD of the original ranks were 20.50 and 11.54, respectively.

^b^ z‐Transformed to a mean of zero and a SD of one; mean and SD of the original ranks were 2.49 and 1.12, respectively.

#### 3.1.2 Model predicting Player 1's choice

We next considered the model testing if Player 1's choice of *Stag* was influenced by Player 1's maximum reward and if Player 2 chose *Stag* in the previous trial. This model (Tables 4 and S3) was not significantly different from the null model (likelihood ratio test comparing full and null model: *χ* ^2^ = 7.53, *df* = 8, *p* = 0.48), suggesting, as in the model of Player 2's choices, that variables where *p* < 0.05 were likely due to Type I error due to multiple testing. There was also no significant effect of the four‐way interaction of Player 2's previous choice, Player 1's maximum reward payout, trial number, and block number (*b *= −0.07, SE = 0.22, *p* = 0.76). In summary, there was no effect of Player 2's previous choice or Player 1's maximum reward payout on Player 1's choice.

**Table 4:** Player 1's choice of Stag predicted by Player 2's choice in the previous trial and Player 1's maximum reward payout

Term	*b*	SE	CI	*p*
Intercept	1.53	0.69	(0.36, 2.29)	
Player 2's previous choice (Stag)	1.31	0.61	(0.28, 2.40)	**0.032**
Trial number^a^	−0.42	0.58	(−1.42, 0.55)	0.47
Block number^b^	−1.77	1.37	(−3.22, −0.00)	0.20
Player 1's maximum payout	0.42	0.22	(0.07, 0.89)	0.052
Player 2's previous choice (Stag): Trial number	0.30	0.64	(−0.72, 1.33)	0.64
Player 2's previous choice (Stag): Block number	1.35	0.60	(0.35, 2.54)	**0.024**
Trial number: Block number	−0.07	0.58	(−1.09, 0.97)	0.90
Player 2's previous choice (Stag): Player 1's maximum payout	−0.40	0.23	(−0.91, −0.03)	**0.075**
Trial number: Player 1's maximum payout	0.22	0.21	(−0.15, 0.57)	0.30
Block number: Player 1's maximum payout	0.77	0.47	(0.15, 1.30)	0.10
Player 2's previous choice (Stag): Trial number: Block number	−0.07	0.60	(−1.12, 0.93)	0.90
Player 2's previous choice (Stag): Trial number: Player 1's maxium payout	−0.15	0.22	(−0.52, 0.24)	0.51
Player 2's previous choice (Stag): Block number: Player 1's maxium payout	−0.45	0.22	(−0.90, −0.06)	**0.041**
Trial number: Block number: Player 1's maxium payout	0.12	0.21	(−0.24, 0.48)	0.56
Player 2's previous choice (Stag): Trial number: Block number: Player 1's maxium payout	−0.07	0.22	(−0.42, 0.32)	0.76

*Note:* Bold values were significant at *p* < 0.05.

Abbreviation: CI, confidence interval.

^a^ z‐Transformed to a mean of zero and a standard deviation (SD) of one; mean and SD of the original ranks were 20.50 and 11.54, respectively.

^b^ z‐Transformed to a mean of zero and a SD of one; mean and SD of the original ranks were 2.49 and 1.12, respectively.

#### 3.1.3 Model predicting self‐scratching behavior

Considering the model testing if self‐scratching behavior was associated with maximum reward payout, we found this model (Table 5; estimated SD of random effects of model in Table S4) was not significantly different from the null model (likelihood ratio test comparing full and null model: *χ* ^2^ = 1.28, *df* = 2, *p* = 0.53). There was also no significant effect of the interaction of block number and maximum reward payout (*b *= −0.15, SE = 0.15, *p* = 0.32). In summary, the maximum reward payout did not influence the amount of self‐scratching the capuchins performed.

**Table 5:** Self‐scratching frequency predicted by maximum payoff

	*b*	SE	CI	*p*
Intercept	−4.51	0.21	(−4.89, −4.09)	
Block number^a^	−0.12	0.08	(−0.25, 0.02)	0.13
Maximum payout^b^	−0.02	0.09	(−0.16, 0.12)	0.78
Block number: maximum payout	−0.15	0.15	(−0.42, 0.14)	0.32

Abbreviation: CI, confidence interval.

^a^ z‐Transformed to a mean of zero and a standard deviation (SD) of one; mean and SD of the original ranks were 2.49 and 1.12, respectively.

^b^ z‐Transformed to a mean of zero and a SD of one; mean and SD of the original ranks were 2.96 and 1, respectively.

### 3.2 Pellet results

**Figure 3:**
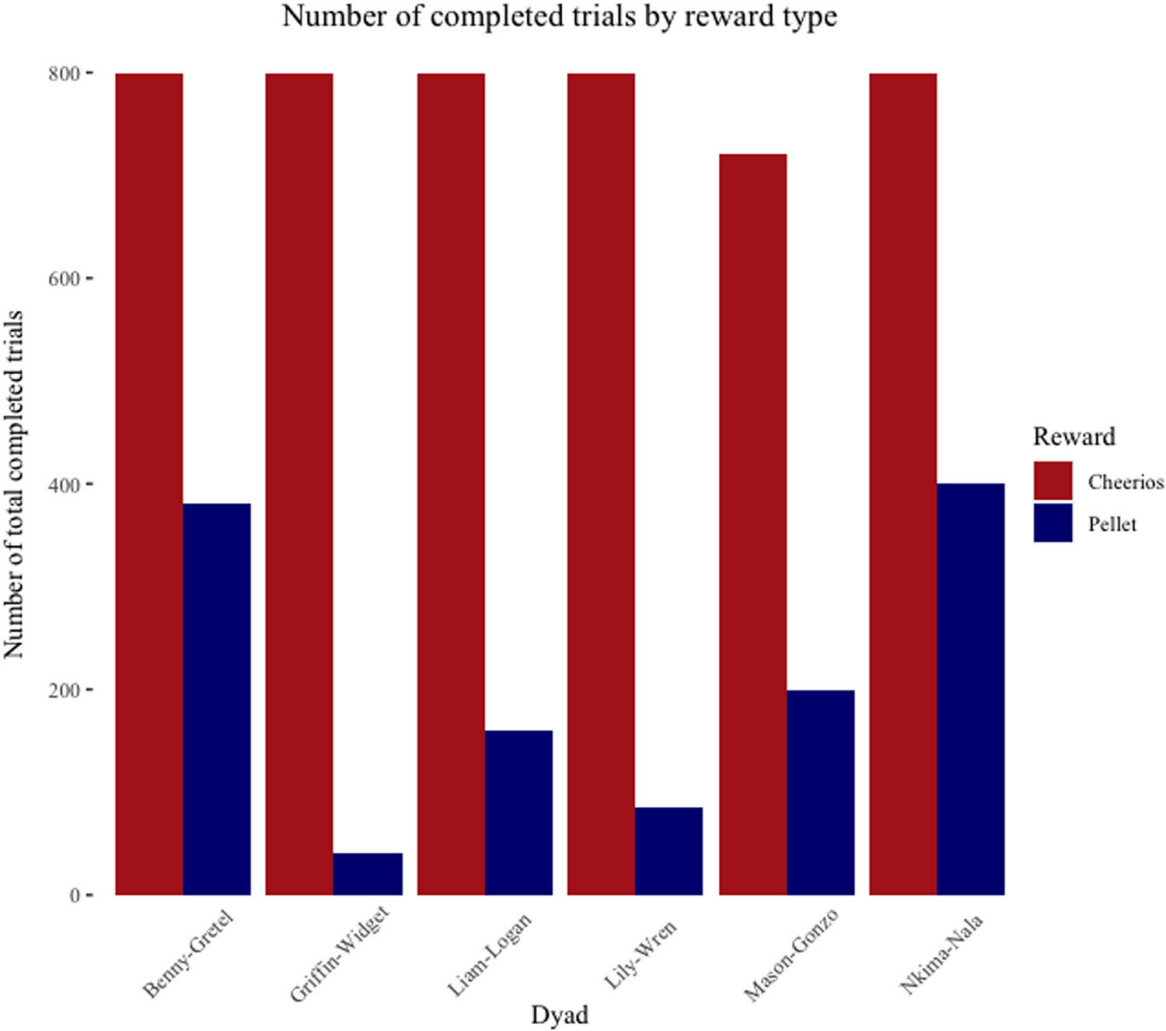
Number of completed trials by reward type

**Figure 4:**
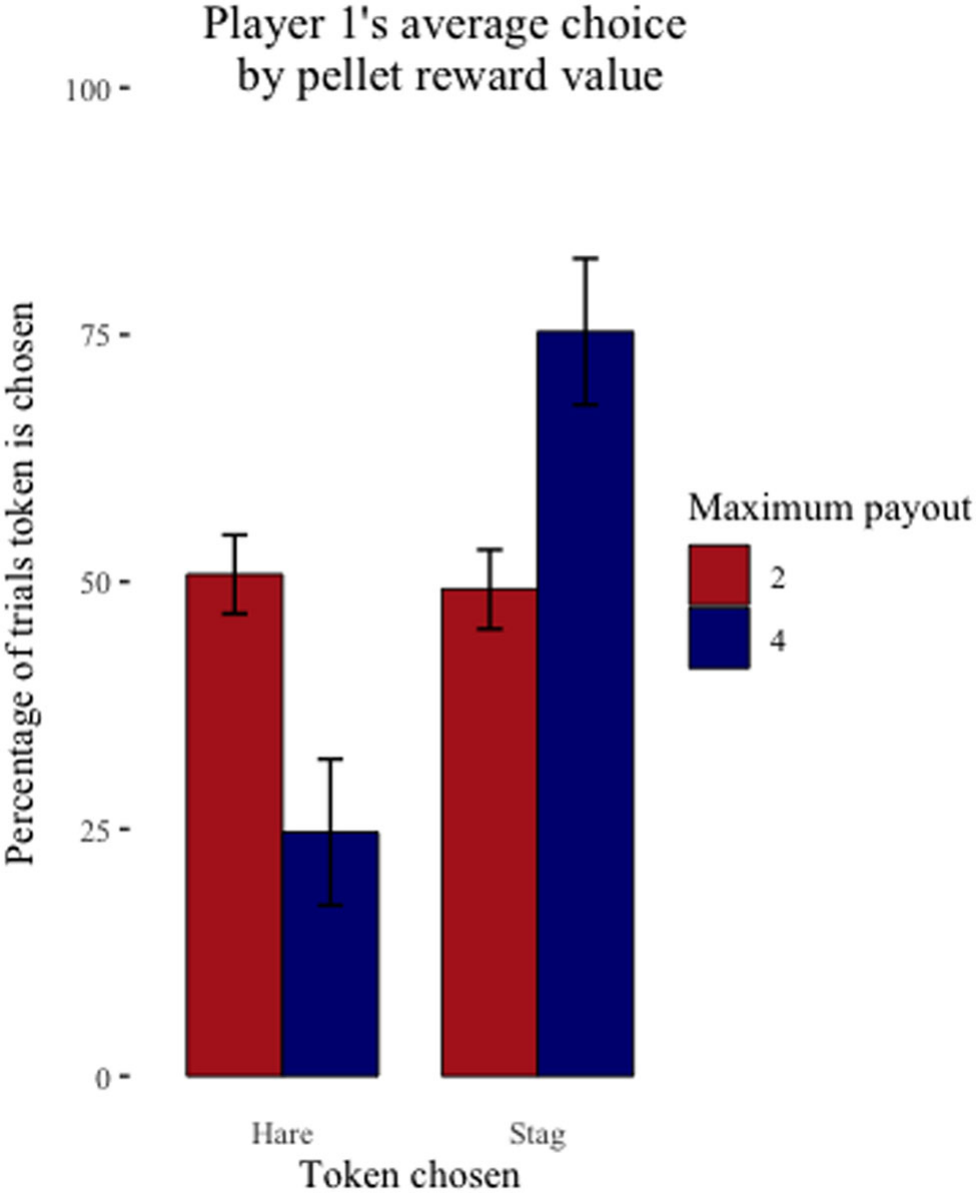
Player 1's average individual choice (Stag or Hare) ± SE by their maximum payout for coordinating on Stag/Stag when pellets are the reward

**Figure 5:**
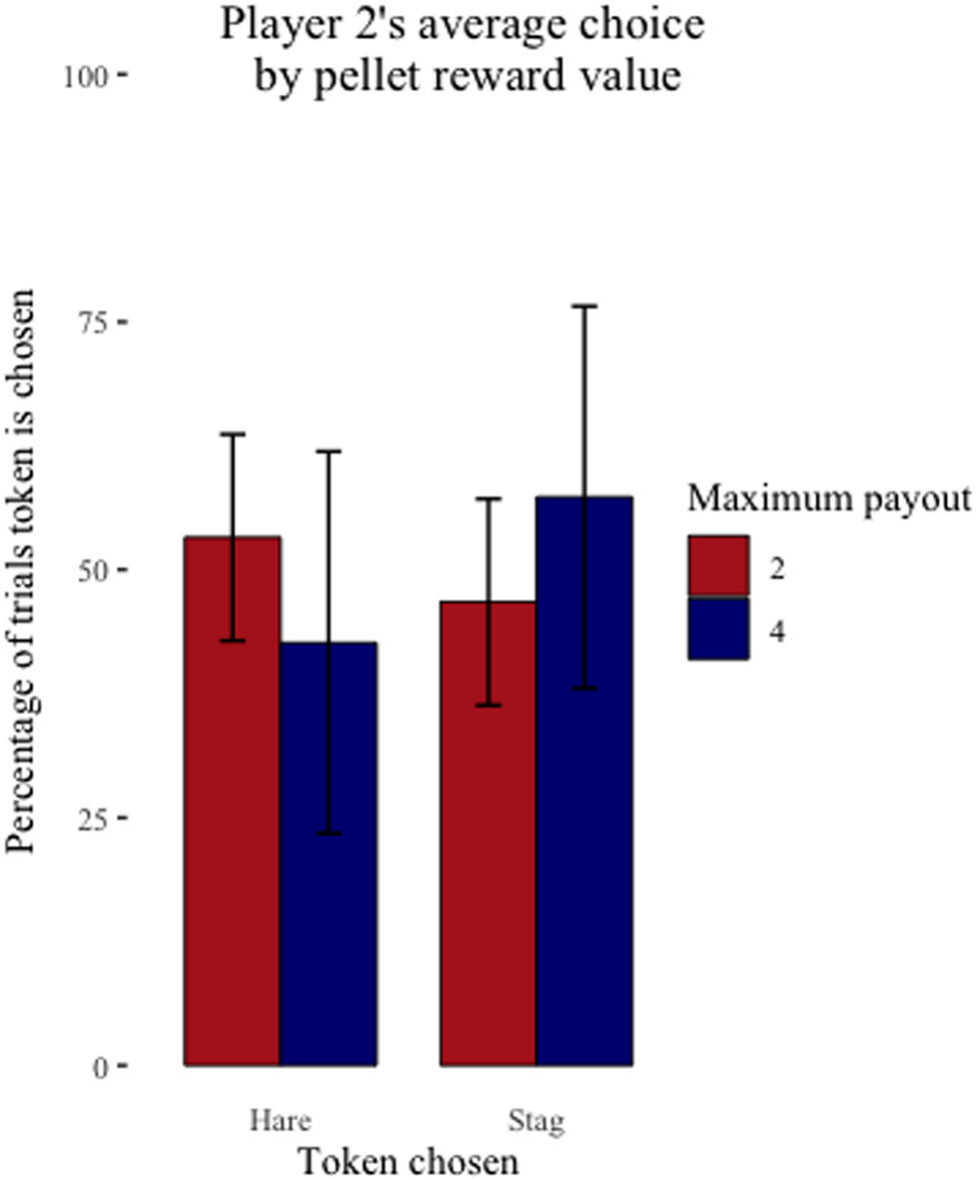
Player 2's average individual choice (Stag or Hare) ± SE by their maximum payout for coordinating on Stag/Stag when pellets are the reward

Overall, the capuchins completed 1266 of 4800 maximum possible trials (26%) when pellets were the reward compared to 4719 of 4800 maximum possible trials (98%) when cheerios were the reward. On average, each dyad completed 211 (SD ± 149.58) pellet trials compared to 786.50 (SD ± 32.58) cheerio trials (Figure 3); note that the number of trials completed varied by each dyad and individual. Of the pellet trials, the capuchins chose to coordinate on the payoff dominant Nash equilibrium (*Stag*/*Stag*) 491 (40%) times; they chose to play the risk dominant Nash equilibrium (*Hare*/*Hare*) 175 (14%) times; they chose the uncoordinated outcome (*Stag*/*Hare* or *Hare*/*Stag*) 600 (47%) times. On average, Player 1 chose *Stag* over *Hare* 49% of the time when their maximum payout was two pellets and 75% of the time when their maximum payout was four pellets (Figure 4). Player 2 chose *Stag* over *Hare* 47% of the time when their maximum payout was two pellets and 57% of the time when their maximum payout was four pellets (Figure 5).

#### 3.2.1 Poisson model predicting Player 1's choice with pellets as the reward

Player 1 chose *Stag* significantly more when they received four pellets instead of two (Table 6; estimated SD of random effects of model in Table S5); there was no significant effect of Player 2's previous choice. This model was significantly different from the null model (likelihood ratio test comparing full and null model: *χ* ^2^ = 11.52, *df* = 2, *p* = 0.009).

**Table 6:** Player 1's choice of Stag predicted by Player 2's choice and Player 1's maximum reward payout with pellets as a reward

	*b*	SE	CI	*p*
Intercept	−0.61	0.09	(−0.80, −0.44)	
Player 2's previous choice (Stag)	0.09	0.09	(−0.09, 0.27)	0.31
Player's 1 maximum payout^a^	0.22	0.09	(0.04, 0.40)	**0.021**
Player 2 previous choice (Stag): Player 2's maximum payout	−0.03	0.10	(−0.22, 0.17)	0.78

*Note*: Bold values were significant at *p* < 0.05.

Abbreviation: CI, confidence interval.

^a^ z‐Transformed to a mean of zero and a standard deviation (SD) of one; mean and SD of the original ranks were 3.11 and 1.02, respectively.

#### 3.2.2 Poisson model predicting Player 2's choice with pellets as the reward

Player 2 chose *Stag* significantly more when they received four pellets instead of two (Table 7; estimated SD of random effects of model in Table S6); there was no significant effect of Player 1's choice. This model was significantly different from the null model (likelihood ratio test comparing full and null model: *χ* ^2^ = 11.50, *df* = 2, *p* < 0.009).

**Table 7:** Player 2's choice of Stag predicted by Player 1's choice and Player 2's maximum reward payout with pellets as a reward

	*b*	SE	CI	*p*
Intercept	−0.80	0.30	(−1.37, −0.26)	
Player 1's choice (Stag)	0.05	0.07	(−0.10, 0.22)	0.48
Player's 2 maximum payout^a^	0.21	0.06	(0.05, 0.38)	**0.001**
Player 1 choice (Stag): Player 2's maximum payout	−0.05	0.08	(−0.22, 0.12)	0.52

*Note:* Bold values were significant at *p* < 0.05.

Abbreviation: CI, confidence interval.

^a^ z‐Transformed to a mean of zero and a standard deviation (SD) of one; mean and SD of the original ranks were 2.89 and 1.02, respectively.

#### 3.2.3 Bayesian model predicting Player 1's choice based on reward value

**Figure 6:**
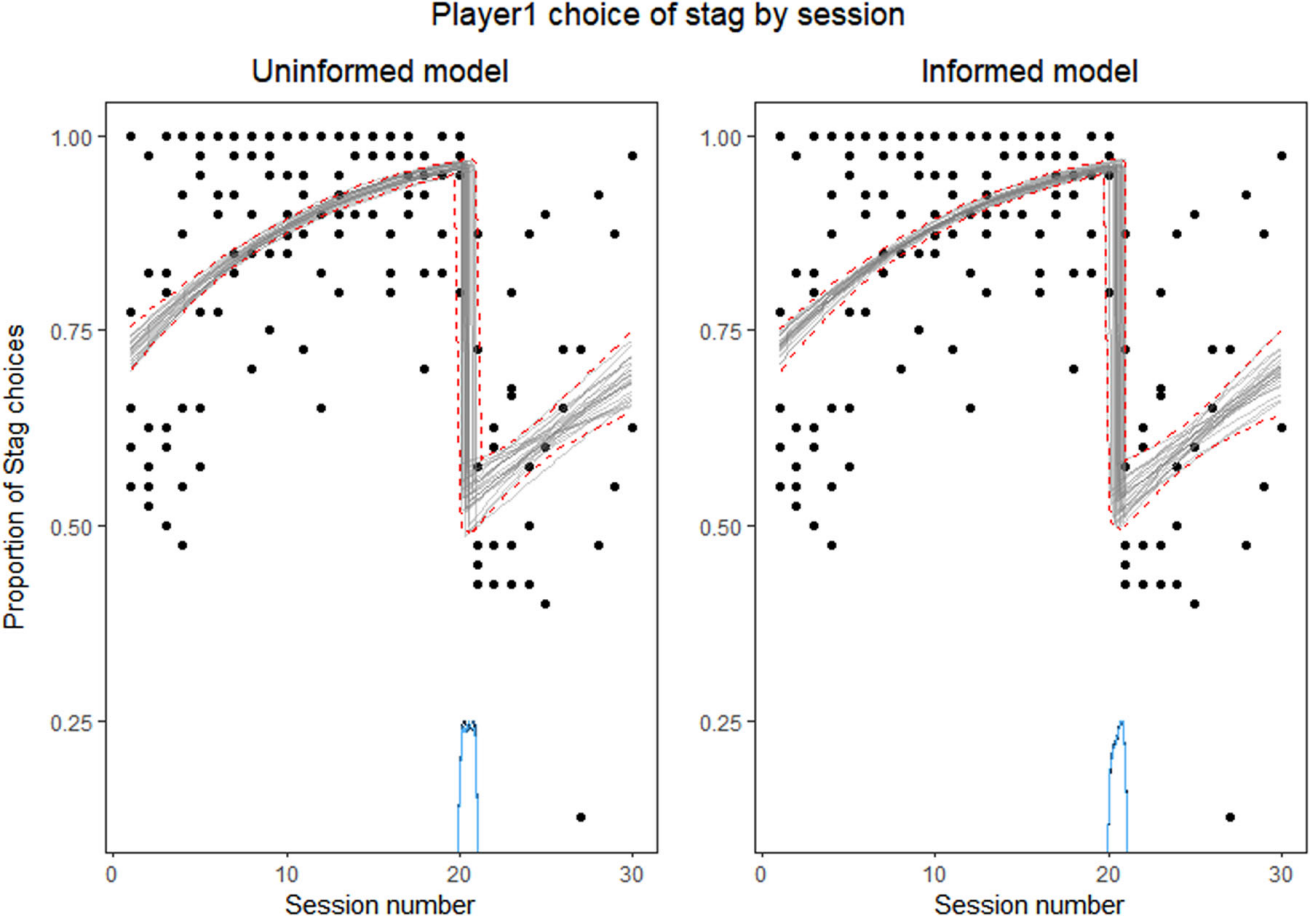
Inferred model of posterior fit of the uninformed (left) and informed (right) model of the proportion of *Stag* choices of Player 1 by session number. Raw data (black dots). Twenty‐five draws from the joint posterior (gray lines). Dashed red lines (95% highest‐density interval). Blue density line (posterior distribution of the change point)

The informed Bayesian regressions with change point showed that there was a change in the proportion of *Stag* choices of Player 1 through the sessions. When comparing the informed model with the null model lacking any change, the informed model (with previous expectations on the exact location of the change point) showed a higher predictive performance (ESLD difference = −300.40). When comparing the informed with the uninformed model, both showed an almost identical predictive performance, with the uninformed model being slightly better (ESLD difference = 0.20). We therefore used the uninformed model to test if the change point was located between the 20th and the 21st sessions. The Bayes factor for the hypothesis stating that the change point is located between the 20th and the 21st session tends to be infinite, as the proportion of explained variance increases to one (Heck, 2019). This indicates strong evidence in favor of our hypothesis (Lee & Wagenmakers, 2014), thus we can conclude that capuchin monkeys participating as Player 1 changed their pattern of choice when they played with pellets as rewards. Specifically, the uninformed model found the change point to be in the point 20.500 (high‐density interval = 20.043–20.990; see Figure 6). Given the small difference in ESLD between the model and the uninformed model, we also checked if the informed model found a similar change point, which it did (change point = 20.542, high‐density interval = 20.068–21.000).

#### 3.2.4 Bayesian model predicting Player 2's choice based on reward value

**Figure 7:**
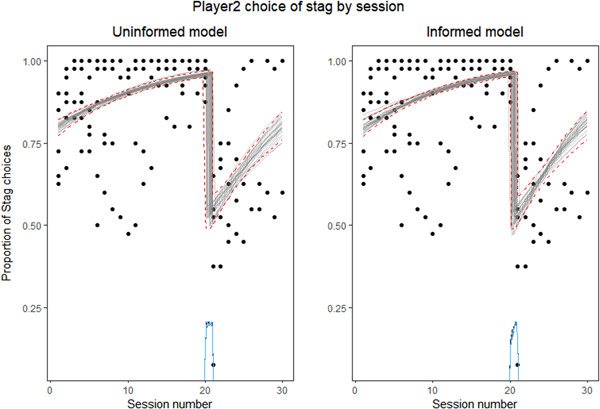
Inferred model of posterior fit of the uninformed (left) and informed (right) model of the proportion of *Stag* choices of Player 2 by session number. Raw data (black dots). Twenty‐five draws from the joint posterior (gray lines). Dashed red lines (95% highest density interval). Blue density line (posterior distribution of the change point)

In the case of Player 2, the comparison between the informed and the null model also favored the informed model (ESLD difference = −246.80), indicating the existence of a change point. When comparing the informed with the uninformed model, both showed almost the same predictive performance, with the uninformed model being slightly better (ESLD difference = −0 to 1). We therefore used the uninformed model to test if the change point was located between the 20th and the 21st sessions. The Bayes factor for this hypothesis tends to be infinite, which indicates that capuchin monkeys participating as Player 2 changed their pattern of choices when the change in reward was introduced. Specifically, the uninformed model found the change point to be in the point 20.497 (high‐density interval = 20.010–20.96; see Figure 7). The informed model also found a similar change point (change point = 20.542, high‐density interval = 20.068–21.00).

Taking these results together, we can infer that the change in reward that occurred after Session 20 affected the performance of the subjects. Subjects playing both as Player 1 or as Player 2 suddenly dropped in the proportion of *Stag* choices when the reward was changed from cheerios to pellets.

## 4 DISCUSSION

Previous experimental studies of cooperation have tended to focus on situations in which rewards were equal for both parties and have often found that cooperation rates drop off when rewards are not evenly distributed (Brosnan et al., 2006; Brosnan & de Waal, 2003; Campbell et al., 2020; Cronin & Snowdon, 2008; de Waal & Davis, 2003). However, these were typically tasks in which subjects had to expend effort to work together, such as pulling in a counterweighted tray to obtain food rewards. We explored whether capuchins would show the same pattern when coordinating in an economic game task, in which subjects must choose one of two options and are rewarded according to what they and their partner choose; earlier work suggested that capuchins are generally very good at finding the coordinated outcome when rewards are equal. Contrary to the earlier findings suggesting that inequality impacts cooperation, in the economic game we found that capuchin monkeys continued to coordinate at very high rates, nearly 80% of the time, despite one individual receiving twice the rewards of the other when rewarded with a preferred reward. We also explored whether subjects would, like humans and dogs, show behavioral signs of distress at inequality in the form of displacement behavior, in this case self‐scratching, despite not refusing to coordinate. However, we found no relationship between self‐scratching and sessions in which the partner received increased rewards.

It is not surprising that capuchins were able to coordinate on the higher‐paying *Stag* equilibrium, as they have done so in many previous studies (Brosnan, Parrish, et al., 2011; Brosnan et al., 2012; Smith et al., 2019). What is surprising is that they continued to do so despite the inequality, which negatively impacts cooperation in other contexts. Indeed, the capuchins in this study coordinated at a *higher* rate than capuchins in the earlier manual task (Brosnan, Parrish, et al., 2011). Better performance itself is not surprising, as these monkeys have gained substantially more experience with economic games since that time, and previous work in chimpanzees suggests that experience appears to improve performance in economic games (Brosnan, Parrish, et al., 2011). It is likely that the capuchins have learned how to maximize their outcomes in these sorts of tasks. Indeed, this is one possible explanation for their lack of a response to the inequality; perhaps they have learned to look for the strategy that maximizes their outcome and stick to that strategy irrespective of other elements of the task. If their main goal is to maximize their food intake, this is a robust strategy and would result in them ignoring the inequality of outcome, as we found.

Another possible explanation is that the capuchins are less sensitive to differences in quantity than quality. In the current task, the rewards differed in quantity, whereas inequality aversion studies usually use differences in quality (Brosnan et al., 2013; Talbot et al., 2016). In a previous study, that included four capuchins who completed this study, Talbot et al. (2018), presented capuchins with a token exchange task in which the animals could first see their partner receiving the same or a better reward for performing the same token exchange, following which they completed the exchange for the reward. Most monkeys were more likely to refuse to participate if their partners received a more preferred food than they did as compared to when their partner got the same reward as long as they were giving up a relatively less preferred food when they refused. However, this was not the case in two situations. First, monkeys never refused if they were receiving a high value food, even if their partner got an even more preferred one. Second, monkeys never refused cheerios, even if their partner got a greater number of them (this was the study that suggested to us a need to rerun the task using pellets instead of cheerios). In all of these studies, every monkey passed a quantity discrimination test before testing, so we know that they preferred more to fewer when given a direct choice. However, subsequent preference tests demonstrated that cheerios were highly preferred, to the same degree as grapes for many capuchins. Thus, one possible explanation is that the cheerios were of such high value reward that the monkeys did not care if their partner was getting more of them. Another possibility is that they do not care about differences in quantity (indeed, this may be particularly relevant when animals are 18″ apart, which may make it difficult to discriminate the number of pieces of a food their partner received). For the purposes of the current study, either could be important.

Of course, as we alluded to above, it may simply be that the cheerios were too valuable to reject. Indeed, humans accept greater inequality in the Ultimatum Game when overall payoffs are higher (Cameron, 1999). Our second condition was a post hoc attempt to test this by studying subjects' responses with a lower value food. However, we ran into the opposite problem; they quickly declined to participate at all for the pellets (they happily accept them for computerized testing, including computerized Assurance games, suggesting that their failure to work for them in manual tasks is a contrast effect due to the fact that they expect the more preferred cheerios and fruit rewards). Indeed, only one pair reached even half of the completed trials (400), whereas all but one pair finished all 800 trials when cheerios were the reward. This suggests to us that they were less motivated to pay attention, and less motivated by the possibility to earn rewards, when pellets were the reward.

When they did work, however, they coordinated on *Stag*/*Stag* only 40% of the time, which is still greater than chance (25%, as there are four possible outcomes), but notably less often than the 78% *Stag/Stag* level when cheerios were the reward. Moreover, based on this unfortunately sparse data set, they were also more likely to choose *Stag* when they received four, rather than two, pellets, suggesting that they may be sensitive to inequity—just not enough to sacrifice two cheerios! This highlights a particular challenge for cognitive and behavioral testing, particularly in situations in which the question is how rewards are influencing behavior; it is difficult to find a reward that is sufficiently valuable for it to be worth the subject's time to participate, but not so high that they never refuse it. Taken in combination with the earlier work that inspired us to run the post hoc pellet condition (e.g., Talbot et al., 2018), these results suggest that studies of cooperation, inequity, and possibly other social contexts, such as social learning, must use extreme care when choosing rewards and, when possible, run studies using several different reward values to determine whether a lack of an effect is due to the rewards simply being too good to pass up.

Despite the suggestive data from the pellet condition, we cannot rule out that the monkeys simply did not care that their partners got a little more. Although many species show inequality aversion, they do not refuse in every situation, particularly if they are receiving a preferred food themselves (Talbot et al., 2018). Moreover, as we discussed above, in nature, cooperating does not guarantee equal pay. For example, chimpanzees do not share the spoils of their hunt evenly (Boesch & Boesch, 1989; Samuni et al., 2018). However, this seems unlikely given that in other contexts of experimental cooperation, capuchins do respond when their partners get more (not to mention the results of our own pellet condition). For instance, studies with brown capuchins show that cooperation is impaired when the rewards are clumped (Brosnan et al., 2006) or one individual tends to monopolize them (de Waal & Davis, 2003). Furthermore, they even share (although unevenly) the spoils of the task when these were obtained cooperatively (de Waal & Berger, 2000), which suggests that brown capuchins do take the distributions of cooperatively obtained rewards into account. Thus, we suspect that the most likely explanation is that the cheerios were simply too high value for the capuchins to refuse.

Contrary to our prediction, unequal pay was not associated with increased self‐scratching, which suggests that receiving fewer cheerios than a partner was not stressful for our capuchins, that they did not notice it, or that self‐scratching was not the appropriate measure. Indeed, this is a single measure, and while we know that our monkeys self‐scratch when they are frustrated (Webster & Brosnan, accepted), in the future it would be worth including other approaches (i.e., other behavioral measures, measures of salivary cortisol), especially since both dogs and humans have been shown to display behavioral responses to unequal outcomes even in situations in which they accept the inequality (Brucks et al., 2016, 2017; LoBue et al., 2011; Range et al., 2009). Aside from its importance for cognitive and behavioral research, knowing when inequality causes stress is also important from a welfare perspective.

Our data have the limitations that we have previously discussed. First, the pellet sessions always followed the cheerio sessions, and compared to a design in which some dyads get pellets first and some get cheerios first, it is difficult to know if the differences were due to a contrast effect (Talbot et al., 2018) of going from high value rewards to low value or due to another reason, such as a natural progression of participating in so many sessions of the same paradigm. Second, while we analyzed the results of the pellet condition, we were unable to collect all of the data (which is, admittedly, data in and of itself), and the Bayesian regressions with change points have been recommended to be used as part of pre‐registered analysis plan (Lindeløv, 2020), which is not our case. Finally, we suffered from a problem typical in such studies: A small sample size that makes it difficult to test more complex associations and consider any other factors, such as experience or age. Indeed, although we built a statistical model to test for several factors, it is likely that our model was substantially underpowered.

Overall, our results suggest the relationship between cooperation, coordination, and inequality aversion is context dependent. We show that capuchins who react to getting less than a partner in some contexts are willing to continue coordinating at very high rates in the Assurance game, even when their partner is getting a greater number of rewards than they do, as long as those rewards are preferred, but our pellet condition hints that they might be more likely to choose relative equity over absolute gains when it does not require sacrificing such a preferred reward. While we cannot rule out other factors, we suspect that, as in Talbot et al. (2018), our monkeys' lack of response to inequity is due to the high value of the reward (cheerios). As a broader point, results emphasize the need to pay careful attention to what rewards are used in each test and to consider what effects they may have on subjects' decisions, particularly in contexts such as cooperation and social learning in which the reward itself is an important part of the decision process.

### Open Research Badges

This article has earned an Open Data badge for making publicly available the digitally‐shareable data necessary to reproduce the reported results. The data is available at https://www.ncbi.nlm.nih.gov/.

## Supporting information

Supporting information.

Supporting information.

Supporting information.

Supporting information.

Supporting information.

Supporting information.

Supporting information.

Supporting information.

Supporting information.

## Data Availability

These data have been made publicly available in the Supporting Information Materials Section (Data S1 and S2) and online repository: DOI 10.17605/OSF.IO/H7EMB

## Footnotes

1 A Nash equilibrium is a strategy where a player can do no better by changing their strategy as long as their partner's strategy remains constant (Fudenberg & Tirole, 1991).
