# Supplementary material for: Anything for a cheerio: Brown capuchins (Sapajus [Cebus] apella) consistently coordinate in an Assurance Game for unequal payoffs
Source: Am J Primatol. 2021 Aug 26;83(10):e23321. doi: 10.1002/ajp.23321 (PMC11475490; doi:10.1002/ajp.23321)
Supplement: Supplementary file 1 — Supporting information. [file AJP-83-e23321-s001.pdf]

Open Practices Disclosure

Manuscript Title: Brown capuchins (Sapajus) [Cebus]apella) consistently coordinate in an Assurance Game for unequal payoffs
Corresponding Author: Lauren M Robinson

Articles accepted to the *American Journal of Primatology* after January 1, 2019 are eligible to earn badges that recognize open scientific practices: publicly available data or material. Please read more about the badges in our journal author guidelines, and you can also find information on the Open Science Framework wiki.

[x] Please check this box if you are interested in participating.

To apply for one or more badges acknowledging open practices, please check the box(es) corresponding to the desired badge(s) below and provide the information requested in the relevant sections. To qualify for a badge, you must provide a URL, doi, or other permanent path for accessing the specified information in a public, open-access repository. **Qualifying public, open-access repositories are committed to preserving data, materials, and/or registered analysis plans and keeping them publicly accessible via the web in perpetuity.** Examples include the Open Science Framework (OSF) and the various Dataverse networks. Hundreds of other qualifying data/materials repositories are listed at http://re3data.org/. **Personal websites and most departmental websites do not qualify as repositories.**

Authors who wish to publicly post third-party material in their data or materials must have the proper authority or permission agreement in order to do so.

There are circumstances in which it is not possible or advisable to share any or all data, materials, or a research plan publicly. For example, there are cases in which sharing participants' data could violate confidentiality. If you would like your article to include an explanation of such circumstances and/or provide links to any data or materials you have made available—even if not under conditions eligible to earn a badge—you may write an alternative note that will be published in a note in the article**.** Please check this box if you would like your article to include an alternative note and provide the text of the note below:

[] Alternative note:

[x] Open Data Badge

1. Provide the URL, doi, or other **permanent path** for accessing the data in a **public, open-access repository**:

https://osf.io/h7emb/

[] Confirm that there is sufficient information for an independent researcher to reproduce **all of the reported results**, including codebook if relevant.

[] Open Materials Badge

1. Provide the URL, doi, or other **permanent path** for accessing the materials in a **public, open-access repository**:

[] Confirm that there is sufficient information for an independent researcher to reproduce **all of the reported methodology**.

By signing below, authors affirm that the above information is accurate and complete, that any third-party material has been reproduced or otherwise made available only with the permission of the original author or copyright holder, and that publicly posted data do not contain information that would allow individuals to be identified without consent.

Signature:
Name: Lauren M Robinson
Date: 8/4/2021
