# Supplementary material for: Anything for a cheerio: Brown capuchins (Sapajus [Cebus] apella) consistently coordinate in an Assurance Game for unequal payoffs
Source: Am J Primatol. 2021 Aug 26;83(10):e23321. doi: 10.1002/ajp.23321 (PMC11475490; doi:10.1002/ajp.23321)
Supplement: Supplementary file 7 — Supporting information. [file AJP-83-e23321-s007.pdf]

Supplementary Table 6 Estimated standard deviations for the contribution of the random effects

Grouping	Effect	SD
Player1	Intercept	0.00
Pair	Intercept	0.69

for the poisson model predicting Player 2's choice of Stag using pellets as a reward
